# PDCD10-Deficient Tumor Cells Reprogram Angiogenesis Through Paracrine Endothelial Activation and GSC-like Plasticity in GBM

**DOI:** 10.3390/cells15171532

**Published:** 2026-08-25

**Authors:** Zhong-Rong Chen, Zhen Chen, Xue-Yan Wan, Maike Anna Busch, Nicole Dünker, Laurèl Rauschenbach, Ulrich Sure, Yuan Zhu

**Affiliations:** 1 Department of Neurosurgery and Spine Surgery, University Hospital Essen, University of Duisburg-Essen, 45147 Essen, Germany; zhong-rong.chen@uk-essen.de (Z.-R.C.); zhen.chen-neurosurgery@outlook.com (Z.C.); xywan@tjh.tjmu.edu.cn (X.-Y.W.); laurel.rauschenbach@uk-essen.de (L.R.); ulrich.sure@uk-essen.de (U.S.); 2 Center for Translational Neuro- and Behavioral Sciences (C-TNBS), University Hospital Essen, University of Duisburg-Essen, 45147 Essen, Germany; 3 Department of Neuroanatomy, Medical Faculty, Institute of Anatomy II, University of Duisburg-Essen, 45147 Essen, Germany; maike.busch@uk-essen.de (M.A.B.); nicole.duenker@uk-essen.de (N.D.)

**Keywords:** glioblastoma, programmed cell death 10, angiogenesis, glioma stem cells, cell plasticity, tumor-derived endothelial cell

## Abstract

Glioblastoma (GBM) is characterized by extensive neo-angiogenesis, which drives rapid tumor growth and therapeutic resistance. We previously identified PDCD10 as a tumor suppressor in GBM. Here, we investigated whether PDCD10 loss promotes neo-angiogenesis through paracrine signaling and GBM cell plasticity. PDCD10 knockdown (shPDCD10) enhanced endothelial angiogenic activity after treatment with conditioned medium (CM) from shPDCD10 cells and in a direct co-culture model. Moreover, application of CM to the chicken chorioallantoic membrane increased vascular branching in an in vivo angiogenesis model. Antibody array analysis detected elevated levels of multiple pro-angiogenic factors following PDCD10 depletion. In a GBM mouse model, shPDCD10 tumors exhibited pronounced hypervascularity and stromal expansion. Indeed, immunofluorescence revealed colocalization of CD31 with the tumor cell reporter RFP in a subset of implanted shPDCD10 GBM cells, suggesting that these tumor cells acquired an endothelial molecular signature. PDCD10 loss also promoted a glioma stem cell (GSC)-like phenotype, characterized by enhanced clonogenicity, sphere formation, and upregulation of Nestin, KLF4, and SOX2. Under endothelial induction conditions, shPDCD10 sphere-derived cells exhibited endothelial-like characters, showing greater tube-forming capacity and increased Ac-LDL uptake. Taken together, these findings demonstrate that PDCD10 loss promotes GBM neo-angiogenesis involving complementary paracrine and GSC-like plasticity mechanisms, highlighting PDCD10 as a potential therapeutic target to suppress neo-angiogenesis in GBM.

## 1. Introduction

Glioblastoma (GBM) is the most common and aggressive malignant primary brain tumor in adults [1,2]. Current standard therapy includes maximal safe surgical resection followed by radiotherapy with concomitant and adjuvant temozolomide. Nevertheless, the median overall survival of GBM patients remains approximately 15 months [3]. Aberrant neo-angiogenesis is a typical pathological feature of GBM and contributes to rapid tumor growth, invasion, hypoxia, blood–brain barrier dysfunction and treatment resistance [4]. The mechanism underlying this vascular phenotype is complex and not yet fully understood. In addition to classical hypoxia-driven angiogenesis, GBM vascularization involves heterogeneous mechanisms, including vessel co-option [5], vasculogenic mimicry, tumor-secreted angiogenic mediators [6,7], host endothelial cells, pericytes, extracellular matrix remodeling and the perivascular stem cell niche [8]. It is known that a fraction of GBM cells displays stem-like properties and is referred to as glioma stem cells (GSCs). GSCs residing in perivascular and hypoxic niches [9,10] may secrete angiogenic factors [11], remodel the tumor microenvironment, and contribute directly to tumor vascularization by differentiating into tumor-derived endothelial cells (TDECs) [12,13] and pericyte-like cells [12], thereby creating tumor-derived vascular elements that blur the boundary between tumor and host endothelium. This vascular plasticity and redundancy contribute to resistance to anti-angiogenic therapy [13] and may also explain why bevacizumab improves radiographic response and progression-free survival but has not consistently improved overall survival [14,15]. Thus, characterization of novel therapeutic targets against abnormal neo-angiogenesis is an emerging strategy for overcoming the adaptive, therapy-resistant vasculature in GBM.

Programmed cell death 10 (PDCD10), originally named TFAR15 (TF-1 cell apoptosis-related gene [16]), is an evolutionarily conserved protein expressed across multiple cell types and human tissues. It is an important regulator of endothelial homeostasis [17]. PDCD10 is also known as cerebral cavernous malformation 3 (CCM3), because loss-of-function mutations in PDCD10 cause familial cerebral cavernous malformation (CCM) [18,19] through dysregulation of multiple pathways, such as RhoA–ROCK [20], DLL4–Notch [21] and MEKK3–KLF2/4 signaling [22]. In endothelial cells, PDCD10 supports cell–cell junction integrity, cytoskeletal organization [20], vesicle trafficking [23,24] and angiogenic signaling [21,24]. Its loss disrupts these processes, leading to increased vascular permeability [20] and abnormal endothelial sprouting [21].

Unlike its established roles in vascular biology and in CCM, PDCD10 shows context-dependent oncological functions, either tumor-promoting or tumor-suppressive effects depending on the cancer type. PDCD10 functions as a tumor promoter in ovarian, prostate and pancreatic cancers [25,26,27]. Data concerning PDCD10 in malignant brain tumors remain limited. Our group reported grade-dependent downregulation of PDCD10 in primary gliomas. Moreover, PDCD10 expression in infiltrative tumor cells and proliferating endothelial cells correlated inversely with tumor cell proliferation and microvessel density in human GBM [28]. We subsequently investigated PDCD10-dependent tumor–endothelial interaction in both in vitro and in vivo models. Endothelial PDCD10 knockdown activated GBM cells through secretion of multiple growth factors [29], whereas PDCD10 loss in GBM cells enhanced malignancy of tumor cells via activation of EphB4 forward signaling [30] and caused temozolomide chemotherapy resistance and rapid tumor cell regrowth by altering apoptosis signaling [31] and by inducing GSC-like states [32]. These findings support PDCD10 as a tumor suppressor in GBM.

Given the absence of PDCD10 in infiltrative tumor cells in hypervascular fields and in proliferating endothelial compartments of human GBM, together with its influence on the secretory profile and chemotherapy resistance [28,29,30,31,32], we hypothesized that PDCD10 deficiency in GBM cells promotes neo-angiogenesis through paracrine endothelial activation and by enabling GBM cell plasticity toward TDEC-like phenotypes. Elucidating these mechanisms may provide insight into how PDCD10 deficiency drives aberrant angiogenesis in GBM.

## 2. Materials and Methods

### 2.1. Lentivirus-Mediated Knockdown of PDCD10 in GBM Cells

Knockdown of PDCD10 was achieved by lentiviral transduction of shRNA in U87 and T98G cells using two distinct vector systems as previously described [29,30,31,32]. Briefly, the inducible TRIPZ lentiviral shRNA vector targeting human PDCD10 (shPDCD10; Thermo Scientific; clone ID: V2THS_217165) was used to transduce U87 cells (shU87). A second lentiviral shRNA vector (OriGene, Rockville, MD, USA; cat# TL302576) was used for stable knockdown of PDCD10 in T98G cells (shT98G). Empty vector-transduced U87 (evU87; Thermo Scientific, Waltham, MA, USA; cat# RHS4750) and T98G (evT98G; OriGene; cat# TR30021) cells served as controls. Red fluorescent protein (RFP) and green fluorescent protein (GFP) served as reporters for ev/shU87 and ev/shT98G cells, respectively. Unless otherwise specified, transduced cells were cultured in Dulbecco’s modified Eagle’s medium (DMEM) supplemented with 10% fetal bovine serum (FBS), 1% sodium pyruvate and puromycin (1 µg/mL; Sigma, Munich, Germany; cat# P8833-25 mg). For the inducible knockdown system in evU87 and shU87 cells, DMEM growth medium was additionally supplemented with doxycycline (dox; 1 µg/mL; Sigma; cat# D9891-10G).

### 2.2. Conditioned Medium Treatment Model

Human umbilical vein endothelial cells (HUVECs) were obtained from PromoCell and cultured in endothelial cell growth medium with supplements (ECGM; cat# C-22010; PromoCell, Heidelberg, Germany). evGBM and shGBM (evU87/shU87 and evT98G/shT98G) cells (3 × 10^6^) were individually plated in a 100 mm Petri dish and cultured in antibiotic-free DMEM growth medium for 3 d. The media were harvested followed by centrifugation at 2000× *g* for 10 min at 4 °C to remove cell debris. The numbers of viable evGBM and shGBM cells were counted using a cell counter at the time of collection. The collected culture medium was then adjusted with fresh DMEM according to the corresponding viable cell number to achieve an equivalent concentration of approximately 5 × 10^6^ viable cells per 10 mL. The conditioned medium (CM) was prepared by mixing 50% of the adjusted culture medium from ev/shGBM and 50% of fresh ECGM. This mixed media were termed evCM and shCM, respectively, and they were used for treatment of HUVECs.

### 2.3. Direct Co-Culture Model

For direct co-culture experiments, HUVECs were directly co-cultured with ev/shT98G cells at a HUVEC/GBM ratio of 1:1 and with ev/shU87 cells at a ratio of 1:4. HUVEC phenotypes were examined after the indicated durations of co-culture.

### 2.4. Examination of Angiogenic Phenotypes in Endothelial Cells (ECs)

Angiogenic phenotypes of endothelial cells (ECs), including proliferation, migration, adhesion, invasion and tube formation, were assessed after treatment with conditioned medium and in a direct co-culture model using previously established protocols with modifications [29,30,33]. In addition, endothelial-like functional properties of tumor-derived endothelial cells (TDECs), including tube formation and acetylated low-density lipoprotein (Ac-LDL) uptake, were evaluated.

Briefly, HUVEC proliferation was detected after 48 h of culture with either evCM or shCM by MTT assay (Invitrogen, Darmstadt, Germany; cat# M6494). For direct co-culture, HUVECs were pre-labeled with CellTrace™ CFSE (8 μM; Thermo Fisher, Waltham, MA, USA; cat# C34554) or CellTrace™ Far Red (8 µM; Thermo Fisher; cat# C34564). Standard curves relating fluorescence intensity to the number of labeled HUVECs were generated using different seeding densities. Fluorescence was measured at excitation/emission wavelengths of 485/535 nm for CellTrace CFSE and 630/661 nm for CellTrace Far Red. After 48 h of co-culture, the fluorescence intensity of labeled HUVECs was measured, and cell numbers were calculated from the corresponding standard curves.

Endothelial adhesion was examined as previously described [29,30], with modifications. HUVECs were suspended in evCM or shCM, seeded into a 96-well plate and incubated for 90 min. Non-adherent cells were removed by gentle washing. Adherent cells were fixed and stained with 0.5% crystal violet and quantified by measuring absorbance at 550 nm using a plate reader.

Endothelial migration was examined by wound healing (scratch) assay. For HUVECs treated with conditioned medium, wounds were recorded at 0 and 24 h after incubation with either evCM or shCM. Wound closure was calculated as the reduction in uncovered wound area at 24 h relative to that at 0 h using ImageJ (version 1.53t). For HUVECs directly co-cultured with evGBM and shGBM cells (direct co-culture model), images were captured by fluorescence microscopy using a 5× objective from six random scratch fields per well in a 6-well plate after 24 h of co-culture in ECGM. RFP-positive ev/shU87 cells and GFP-positive ev/shT98G cells were recorded. To visualize all cells, including HUVECs and GBM cells, DAPI staining was performed. Total migrated cells with DAPI-positive nuclei and migrated RFP- or GFP-positive GBM cells were counted using ImageJ. The number of migrated EC was calculated by subtracting the number of migrated fluorescent GBM cells from the total number of migrated cells within the defined scratch area.

Endothelial invasion was assessed using a Transwell invasion assay. HUVECs (5 × 10^4^ cells per insert in 24-well plates) were suspended in 200 μL supplement-free ECGM and seeded into Matrigel-coated inserts (1 mg/mL; Corning, NY, USA; cat# 356234). For a conditioned medium treatment model, 700 μL of CM was added to the lower chamber. For direct co-culture model, GBM cells (1 × 10^5^ cells) were seeded into the lower chamber and cultured with DMEM. After 24 h of incubation, non-invaded cells on the upper surface of the membrane were removed with a cotton swab. Invaded cells on the underside were fixed with 4% paraformaldehyde, stained with 0.5% crystal violet and quantified in five random fields acquired at 20× magnification.

The endothelial tube formation assay was performed in HUVECs treated with conditioned medium and in TDEC-like cells. HUVECs or TDEC-like cells (2.5 × 10^4^ cells per well) were seeded onto 50 μL Matrigel-precoated 96-well plates. TDEC-like cells were maintained in endothelial cell growth medium-2 (ECGM2; PromoCell, Heidelberg, Germany; cat# C-22011) supplemented with doxycycline (1 μg/mL). Tube formation was examined after 12 h of incubation. Images were acquired from five random fields per well using a 5× objective, and branching points in tube-like structures were quantified using ImageJ.

### 2.5. Ac-LDL Uptake Assay

The Ac-LDL uptake assay was performed according to the manufacturer’s instructions. Briefly, evTDEC-like cells and shTDEC-like cells were seeded into 96-well plates (4000 cells/well) and cultured overnight. The next day, cells were serum-starved for 30 min and then incubated with Alexa Fluor™ 488 Ac-LDL (5 μg/mL; Invitrogen; cat# L23380) for 4 h at 37 °C in a humidified incubator containing 5% CO_2_. Cells were then washed twice with PBS to remove unbound Ac-LDL and counterstained with DAPI (2 μg/mL; Thermo Scientific, Schwerte, Germany). Fluorescence images were acquired using a fluorescence microscope. Ac-LDL uptake was quantified by measuring fluorescence intensity with a plate reader at excitation/emission wavelengths of 485/535 nm.

### 2.6. Angiogenesis Model in the Embryo Chicken Chorioallantoic Membrane (CAM)

The CAM angiogenesis model was performed as previously described [33,34], with modifications. Briefly, fertilized chicken eggs were incubated in a humidified rotary incubator at 38 °C and 50% humidity for 10 d. On embryonic day 10 (ED10), eggs were candled and the chorioallantoic vein was identified. An approximately 1 cm^2^ square near a venous branch was marked, a hole was made in the air sac and a window was opened to access the CAM. CM (400 μL; 50% collected ev/shGBM culture medium and 50% ECGM) was applied to the CAM surface. The eggs were sealed and incubated for a further 72 h. On ED13, CAM vasculature was imaged using a stereomicroscope. Vascular branching points were subsequently quantified in five random fields per CAM using ImageJ software (version 1.53t) [35].

For histological analysis, CAM specimens from the CM-treated area were harvested, fixed in 4% paraformaldehyde, paraffin-embedded and sectioned at a thickness of 4 μm. Microvessels with diameters of 10–50 μm were quantified in 10 fields per hematoxylin and eosin (H&E)-stained section using a 10× objective.

### 2.7. RNA Extraction, cDNA Synthesis and RT^2^-PCR

Total RNA was extracted using the innuPREP DNA/RNA Mini Kit (Analytik Jena AG, Jena, Germany). cDNA was synthesized using the iScript cDNA Synthesis Kit (Bio-Rad, Munich, Germany) according to the manufacturer’s instructions. Real-time PCR was performed using RT^2^ SYBR Green/ROX qPCR Mastermix (Qiagen, Hilden, Germany) on a CFX Connect Real-Time PCR Detection System (Bio-Rad). Primer sequences and annealing temperatures used in this study are listed in Table 1. The relative expression of a gene of interest was calculated using the 2^−ΔΔCt^ method, with GAPDH or RPS13 as the reference gene for U87 or T98G, respectively [36].

### 2.8. Western Blotting

Western blotting was performed as previously described [29,30]. The following antibodies were used: rabbit anti-PDCD10 (1:800; Abcam, Cambridge, UK; cat# ab180706), rabbit anti-GAPDH (1:1000; Cell Signaling Technology, Danvers, MA, USA; cat# 2118) and HRP-linked anti-rabbit IgG (1:2000; Cell Signaling Technology; cat# 7074). Signals were detected by chemiluminescence using Clarity ECL substrate (Bio-Rad; cat# 1705060) and an ImageQuant LAS 500 system (GE Healthcare, Freiburg, Germany). Semi-quantification was performed by measuring blot optical density using ImageJ [35].

### 2.9. Colony Formation Assay

The colony formation assay was performed as previously described [30,32]. GBM cells (500 cells/well) were seeded in a 12-well plate, followed by 13 d of incubation. The growth medium was refreshed every 5–6 d. Thereafter, cells were fixed in 4% paraformaldehyde and stained with 0.5% crystal violet. Stained colonies were imaged using a digital scanner. The number of colonies per well was analyzed using ImageJ software (version 1.54j) [35]. Colony formation efficiency (CFE) was calculated as follows: CFE = (number of colonies/number of seeded cells) × 100%.

### 2.10. Generation of Glioma Stem Cell (GSC)-like Cells by Sphere Formation Assay

To generate GSC-like cells, ev/shU87 and ev/shT98G cells were cultured in GSC medium consisting of DMEM/F12 (Gibco, Grand Island, NY, USA; cat# 11320033) supplemented with 1× B27 (Gibco; cat# 17504044), 2× N2 (Gibco; cat# 17502048), 20 ng/mL recombinant human EGF (PeproTech, Hamburg, Germany; cat# AF-100-15) and 20 ng/mL recombinant human basic FGF (PeproTech; cat# 100-18C). Half of the GSC medium was refreshed every 3–4 d for around 2 wk until spheres formed. Spheres were imaged using LAS X software (version 3.7.6.25997; Leica, Wetzlar, Germany) with a 5 × objective. Sphere formation efficiency (SFE, %) was calculated by counting spheres larger than 50 µm in diameter using the following formula: SFE = (number of spheres/number of seeded cells per well) × 100%. Thereafter, the sphere suspension was harvested and centrifuged at 200× *g* for 3 min. Collected spheres were dissociated into single cells using StemPro Accutase (Gibco; cat# A11105-1). These sphere-derived cells were termed evGSC-like cells or shGSC-like cells and were either expanded further in GSC medium or used in the indicated experiments.

### 2.11. Culture of GSC-like Cells Under Endothelial Induction Conditions

evGSC-like cells or shGSC-like cells were seeded (each 3 × 10^5^) in 60 mm dishes and maintained in endothelial cell growth medium-2 (ECGM2; PromoCell, Heidelberg, Germany; cat# C-22011) supplemented with doxycycline (1 μg/mL). The medium was refreshed every 2 to 3 d for 2 wk. The resulting populations were defined as tumor-derived endothelial-like cells (evTDEC-like cells and shTDEC-like cells). These cells were maintained in ECGM2 from passages 2 to 10 before being used for gene expression analyses and functional assays.

### 2.12. Flow Cytometry

Flow cytometry was performed to evaluate endothelial marker expression in TDEC-like cells. TDEC-like cells (1 × 10^5^) were harvested, washed twice and resuspended in PBS, followed by incubation with fluorophore-conjugated anti-human CD31 (5 μL; BioLegend, San Diego, CA, USA; cat# 303123) or anti-human CD144 (5 μL; Proteintech, Rosemont, IL, USA; cat# CL488-83766-6) in 100 μL PBS for 45 min at 4 °C in the dark. In parallel, a separate aliquot of TDEC-like cells was incubated with the corresponding fluorophore-matched non-specific isotype control to establish background fluorescence for gating. After incubation, cells were washed with PBS to remove unbound antibodies and analyzed using a CytoFLEX instrument (Beckman Coulter, Indianapolis, IN, USA). The percentages of CD31-positive and CD144-positive cells were quantified using FlowJo software (version 10.9.0).

### 2.13. H&E and Elastic van Gieson (EVG) Staining and Immunofluorescence Staining of Xenograft Tumor Sections from a GBM Mouse Model

The xenograft tumor sections were archived material derived from a GBM mouse model. The animal experiments were performed strictly according to the ethics contract approved by the local ethical committee (No. 84-02.04.2012.A348). In this model, evU87 and shU87 cells were implanted into the mouse flank, and xenograft tumors were removed 3 wk after implantation for paraffin embedding and sectioning [30,31]. H&E and elastic van Gieson (EVG) staining were used for histological analysis and visualization of vascular and stromal structures, respectively. Double immunofluorescence staining was performed by incubating sections with a primary antibody mixture containing Alexa Fluor 488-conjugated anti-CD31 (1:200; Abcam; cat# ab215911) and anti-RFP (1:200; Abcam; cat# ab185921). For negative controls, sections were incubated with non-immune rabbit or mouse IgG at the same concentrations as the corresponding primary antibodies. After washing, Texas Red-conjugated anti-rabbit antibodies (1:200; Vector, Newark, CA, USA; cat# TI-1000) were applied, and nuclei were counterstained with DAPI (Thermo Scientific, Schwerte, Germany). Fluorescence images were acquired using an AxioImager M.2 microscope (Carl Zeiss AG, Oberkochen, Germany).

### 2.14. Angiogenesis Protein Array

An angiogenesis protein array was carried out with the culture media from ev/shT98G cells after 72 h of seeding by using a human angiogenesis array kit (R&D Systems, Minneapolis, MN, USA; ARY007) according to the manufacturer’s protocol. To eliminate the influence of cell numbers on the secretory profile, the collected culture media from evGBM and shGBM cells were normalized to the corresponding viable cell numbers before the array. This kit detects 55 angiogenesis proteins using individual specific antibodies precoated in duplicate on blot membranes. The membranes were respectively incubated with collected culture media from evGBM and shGBM cells, and the immunoreactive spots were visualized by chemiluminescent detection with multiple exposure times by using ImageQuant LAS 500 (GE Healthcare, Freiburg, Germany) followed by semi-quantification with the ImageJ software (version 1.53t). The mean optical intensity of duplicate spots was calculated, and data were presented as the fold change in shGBM relative to the corresponding ev control. Upregulation of individual proteins showing more than a 1.5-fold increase in the shGBM group was listed.

### 2.15. Statistics

Statistical analyses were performed using GraphPad Prism 8. Data are presented as mean ± SD unless otherwise stated. Two-group comparisons were analyzed using Student’s *t*-test. Comparisons involving more than two groups were analyzed using one-way ANOVA followed by Scheffé’s multiple-comparison test. Unless otherwise indicated, each independent experiment (biological replicate) was performed at least three times. For cell-based assays, each biological replicate was derived from an independently cultured cell population, and technical replicates within each experiment were averaged before statistical analysis. A two-sided *p* value < 0.05 was considered statistically significant.

## 3. Results

### 3.1. Treatment of Conditioned Medium from PDCD10-Knockdown GBM Cells Enhanced Endothelial Angiogenic Activity

**Figure 1:**
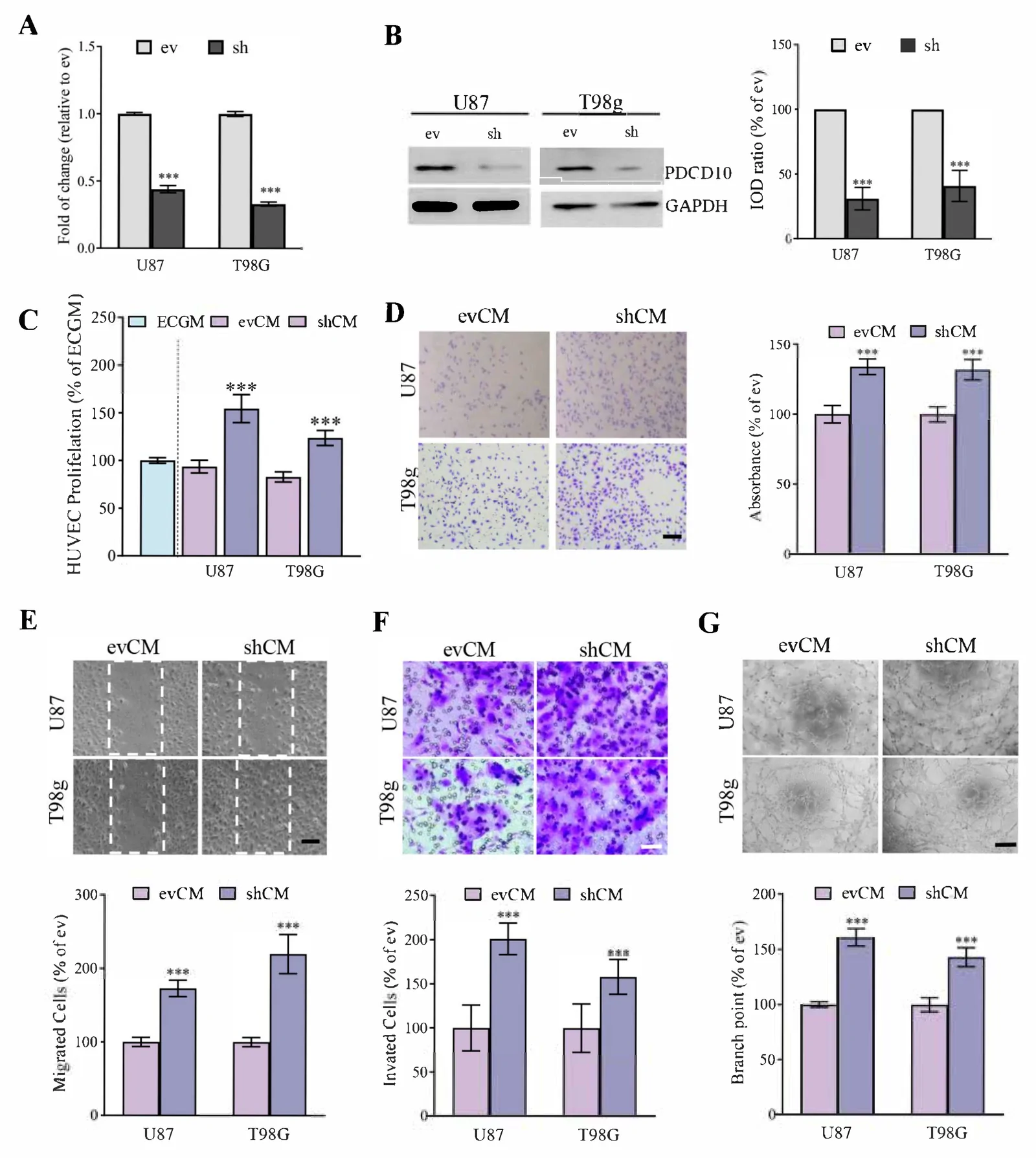
PDCD10 knockdown in GBM cells activated endothelial angiogenesis in the conditioned medium treatment model. PDCD10 was stably knocked down in U87 and T98G cells by lentiviral transduction of shRNA plasmid (sh) or empty vector (ev). HUVECs were cultured in conditioned medium (CM) composed of 50% of the ev/shU87 or ev/shT98G culture medium and 50% of endothelial cell growth medium (ECGM). (**A**) Confirmation of PDCD10 knockdown by RT^2^-PCR analysis. (**B**) Confirmation of PDCD10 knockdown by Western blot. IOD, integrated optical density. (**C**) Cell proliferation assay. HUVEC proliferation was assessed using the MTT assay after culture in ECGM as the reference condition (data were presented in the left side of dash line) or in the respective conditioned medium (CM) derived from U87 and T98G cells(data were shown in the right side of dash line). (**D**) Adhesion assay. HUVEC adhesion significantly increased after incubation with either type of shCM compared with the corresponding evCM. Representative images (left) and the quantitative analysis (right) are shown. (**E**) Migration assay. HUVEC migratory activity markedly increased in the shCM groups. Upper panel: Representative images acquired 24 h after scratching. Lower panel: Quantitative analysis of wound closure. (**F**) Transwell invasion assay. HUVEC invasive activity was significantly enhanced in the shCM groups. Upper panel: Representative images of invaded HUVEC after 24 h. Lower panel: Quantitative analysis based on five random fields. (**G**) Tube formation assay. HUVECs cultured with shCM showed a greater tube formation capacity compared with the corresponding evCM groups. Upper panel: Representative images of capillary-like structures acquired 12 h after seeding on Matrigel. Lower panel: Quantitative analysis of branching points in each field and five randomized fields per well were analyzed. All experiments were independently repeated three times. ***, *p* < 0.001, compared with the corresponding ev or evCM, as indicated. Scale bar: 50 μm in (**D**–**G**).

As detected by RT^2^-PCR, PDCD10 mRNA expression decreased to 44% and 33% of the corresponding empty vector (ev) controls in shU87 and shT98G cells, respectively (both *p* < 0.001; Figure 1A). Western blotting confirmed downregulation of PDCD10 protein expression to 31% and 41% of control levels in shU87 and shT98G cells, respectively (both *p* < 0.001; Figure 1B, uncropped blots are shown in in Supplementary Figure S1). In the conditioned medium treatment model, proliferation of HUVECs increased to 154% and 123% of the ECGM control level after culturing with CM from shU87 or shT98G, respectively (both *p* < 0.001; Figure 1C), whereas HUVECs cultured in the corresponding evCM showed proliferation levels similar to the ECGM control. Consistently, treatment with CM from shU87 and shT98G enhanced other angiogenic behaviors of HUVECs, respectively increasing adhesion to 134% and 132% of the corresponding evCM levels (both *p* < 0.001; Figure 1D), migration by 73% and 120% relative to evCM (both *p* < 0.001; Figure 1E), and invasion to 201% and 158% of the corresponding evCM levels (both *p* < 0.001; Figure 1F). HUVECs formed more tube-like structures after incubation with CM from shU87 and shT98G. Quantitative analysis showed significantly higher numbers of branching points after 12 h of incubation with CM from shU87 and shT98G, reaching 161% and 143% of the corresponding evCM levels, respectively (both *p* < 0.001; Figure 1G).

### 3.2. Direct Co-Culture with PDCD10-Knockdown GBM Cells Enhanced Endothelial Proliferation, Invasion and Migration

**Figure 2:**
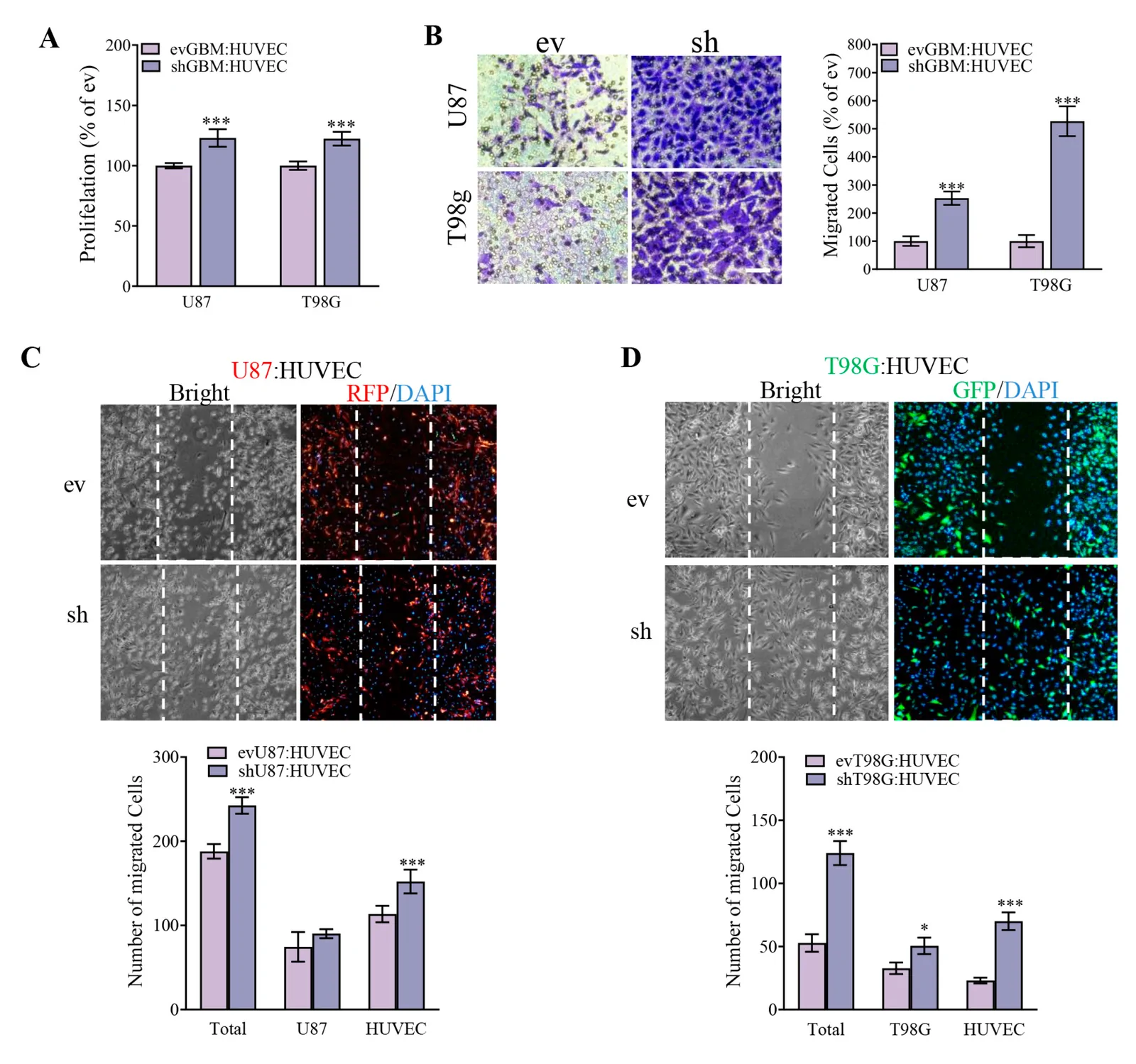
Direct co-culture with PDCD10-knockdown GBM cells enhanced endothelial proliferation, invasion and migration. For direct co-culture, HUVECs were co-cultured with empty vector-transduced (ev) or PDCD10-knockdown (sh) GBM cells in ECGM at ratios of 1:4 and 1:1 for ev/shU87 and ev/shT98G, respectively, followed by examination of endothelial behaviors. (**A**) Proliferation assay. HUVECs were pre-labeled with CellTrace CFSE or CellTrace Far Red. The proliferation of HUVECs was evaluated by detecting the fluorescence intensity of the respective dyes using a plate reader at excitation/emission wavelengths of 485/535 nm for CellTrace CFSE and 630/661 nm for CellTrace Far Red. Direct co-culture with shGBM cells significantly stimulated endothelial proliferation compared with the corresponding ev groups. (**B**) Transwell invasion assay. HUVECs were seeded into the upper Matrigel-coated inserts, whereas ev- or shGBM cells were placed into the lower chambers. Endothelial invasion was detected by crystal violet staining at 24 h of co-culture (left panel). Quantitative analysis of five random fields showed significantly greater endothelial invasion toward either shU87 or shT98G cells compared with the corresponding ev groups (right panel). (**C**,**D**) Migration assay in the co-culture of U87:HUVEC (**C**) and T98G:HUVEC (**D**). Transduced ev/shU87 and ev/shT98G cells carried red fluorescent protein (RFP) or green fluorescent protein (GFP) fluorescence vectors, respectively. Total migrated cells were identified by counting DAPI-positive nuclei. The number of migrated HUVECs was calculated by subtracting migrated fluorescent ev/shGBM cells from total migrated cells. Upper panels: Representative bright-field and fluorescence images acquired 24 h after scratching. Lower panels: Quantitative analysis of migrated HUVECs. All experiments were independently repeated three times. *, *p* < 0.05 and ***, *p* < 0.001, compared with the corresponding ev controls, as indicated. Scale bar: 50 μm.

In the direct co-culture model, HUVECs were co-cultured with ev/shU87 or ev/shT98G cells. For the proliferation assay, HUVEC were pre-labeled with CellTrace CFSE for co-culture with U87 cells or CellTrace Far Red for co-culture with T98G cells. Endothelial proliferation was evaluated by measuring fluorescence intensity in the pre-labeled HUVECs. HUVEC fluorescence intensity increased by 23% and 22% after co-culture with shU87 and shT98G cells, respectively, compared with the corresponding evU87 and evT98G controls (both *p* < 0.001; Figure 2A). In the Transwell invasion assay, HUVECs seeded in the upper inserts exhibited markedly greater invasion toward shU87 and shT98G cells plated in the lower chambers. The numbers of invaded HUVECs were 253% and 527% of those observed with the corresponding evU87 and evT98G cells, respectively (both *p* < 0.001; Figure 2B). In the scratch assay, HUVECs were co-seeded with either RFP-labeled ev/shU87 or GFP-labeled ev/shT98G cells. The left columns of Figure 2C,D show representative bright-field images of total seeded cells, whereas the right columns specifically show RFP-labeled ev/shU87 or GFP-labeled ev/shT98G cells, respectively. For quantitative analysis, the total number of migrated cells and the numbers of migrated RFP-positive ev/shU87 or GFP-positive ev/shT98G cells were counted. HUVEC migration was calculated by subtracting fluorescent GBM cells from the total migrated cell number. The total number of migrated cells and the number of migrated HUVECs were significantly higher in the shU87:HUVEC group than in the evU87:HUVEC group (both *p* < 0.001; Figure 2C). Similar results were obtained in direct co-cultures of HUVECs with ev/shT98G cells (Figure 2D).

### 3.3. Conditioned Medium Derived from PDCD10-Knockdown GBM Cells Promoted Angiogenesis in the Chicken Chorioallantoic Membrane (CAM) Model

**Figure 3:**
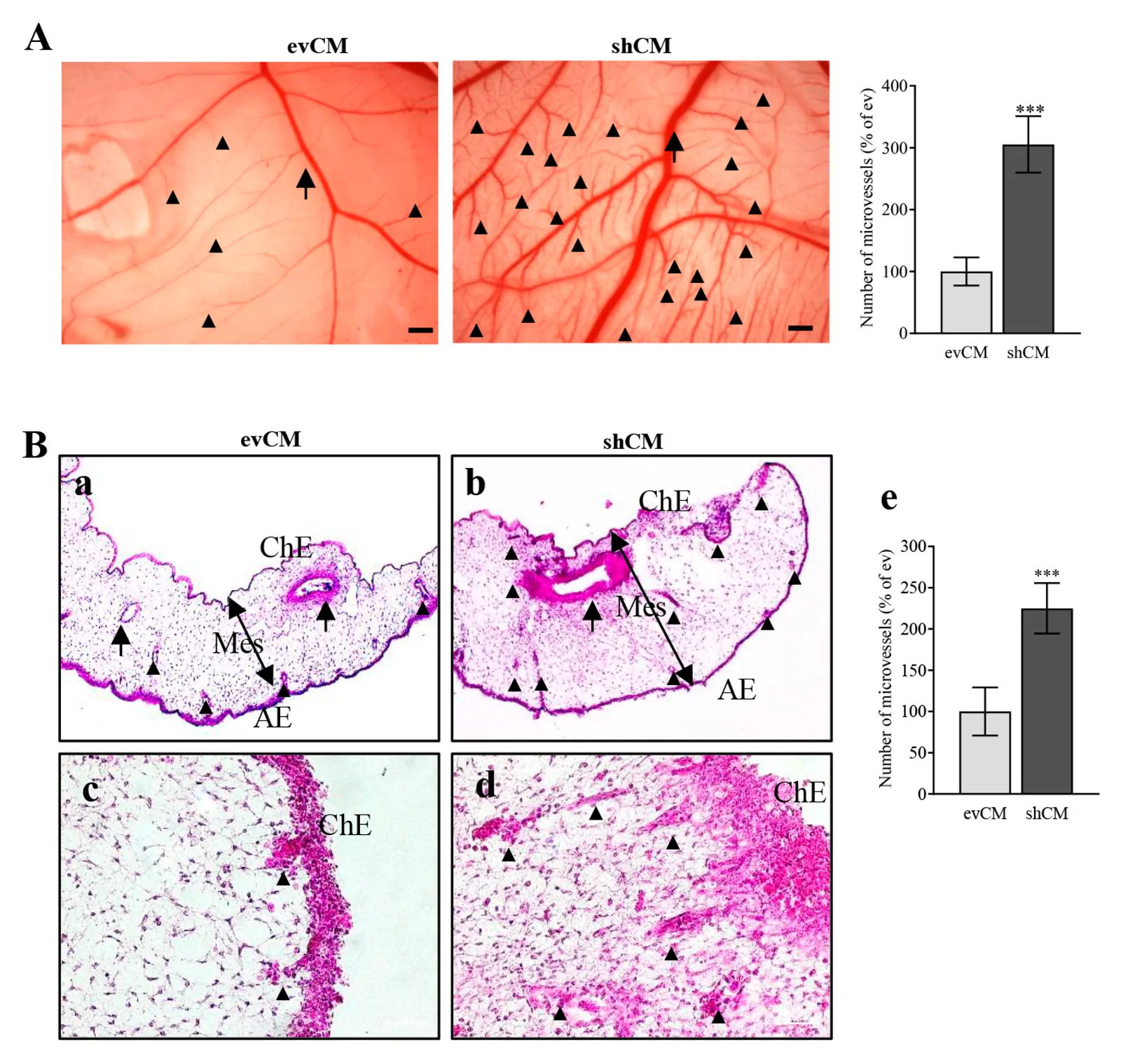
Conditioned medium derived from PDCD10-knockdown GBM cells stimulated angiogenesis in an in vivo angiogenesis model on the chicken chorioallantoic membrane (CAM). Conditioned medium evCM or shCM (each 400 μL), referring to a mixture of 50% culture medium from evT98G and shT98G cells and 50% ECGM, was applied to the CAM on embryonic day 10 (ED10), followed by further incubation for 3 d. CAM vasculature was macroscopically recorded and analyzed on ED13. (**A**) Representative stereomicroscopy images of CAM vasculature after treatment with evCM or shCM (left panel). Large CAM vessels are indicated by arrows, whereas microvessels are indicated by arrowheads. A denser and more highly branched vascular network was observed in the shCM-treated group. Quantitative analysis (right panel) demonstrated a significantly higher number of microvascular branching points in the shCM group compared with the evCM. Data are presented as the mean of five random fields per CAM section in independent replications for evCM group (*n* = 7) and for shCM group (*n* = 6). (**B**) Histological analysis on H&E-stained CAM sections. The CAM consists of multiple layers, including chorionic epithelium (ChE), mesenchymal layer (Mes) and allantoic epithelium (AE). Representative H&E-stained CAM images after treatment with evCM (**B**(**a**,**c**)) or shCM (**B**(**b**,**d**)). Microvessels in the Mes layer iare indicated by arrowheads, whereas larger CAM vessels are indicated by arrows. The high-magnification view revealed significantly more microvessels in the Mes layer of the shCM-treated CAM (**B**(**d**)) compared with the evCM group (**B**(**c**)). Quantitative analysis of microvessels with diameters from 10 to 50 μm indicate a 2.5-fold increase in microvessel density in the shCM group compared with the evCM group (**B**(**e**)). Data are presented as mean of 10 random fields per section in independent CAMs: *n* = 7 for evCM and *n* = 6 for shCM. ***, *p* < 0.001, compared with evCM. Scale bar: 1 mm in (**A**).

Next, we examined whether conditioned medium (evCM and shCM referred to a mixture of 50% culture medium from evT98G and shT98G cells and 50% ECGM) promoted angiogenesis in an in vivo angiogenesis model. evCM or shCM was applied to the CAM on embryonic day 10 (ED10) and vasculature was assessed 72 h later. The images from stereomicroscopy (left panel of Figure 3A) showed larger vessels (arrows) and denser, more highly branched microvessels (arrowheads) in the shCM group than in the evCM group. Quantitative analysis demonstrated that the number of vascular branching points increased to 305% of the evCM level in the shCM group (*p* < 0.001; *n* = 7 for evCM and *n* = 6 for shCM; right panel, Figure 3A). H&E staining revealed multilayered histological structure of CAM: the chorionic epithelium (ChE), mesenchymal layer (Mes) and allantoic epithelium (AE) in the CAM (Figure 3B(a,b)). Bigger vessels (arrow) and microvessels (arrowheads) were clearly visualized in the images from high-magnification microscope views (Figure 3B(c,d)) where more abundant microvessels were visible in the Mes layer in shCM-treated CAM (Figure 3B(b,d)) compared with the control evCM (Figure 3B(c)). Quantitative analysis of microvessels with diameters of 10–50 µm showed a 2.5-fold increase in the shCM group compared with the evCM group (*p* < 0.001; *n* = 7 for evCM and *n* = 6 for shCM; Figure 3B(e)).

### 3.4. PDCD10 Knockdown in T98G Cells Induced a Pro-Angiogenic Secretory Profile

**Figure 4:**
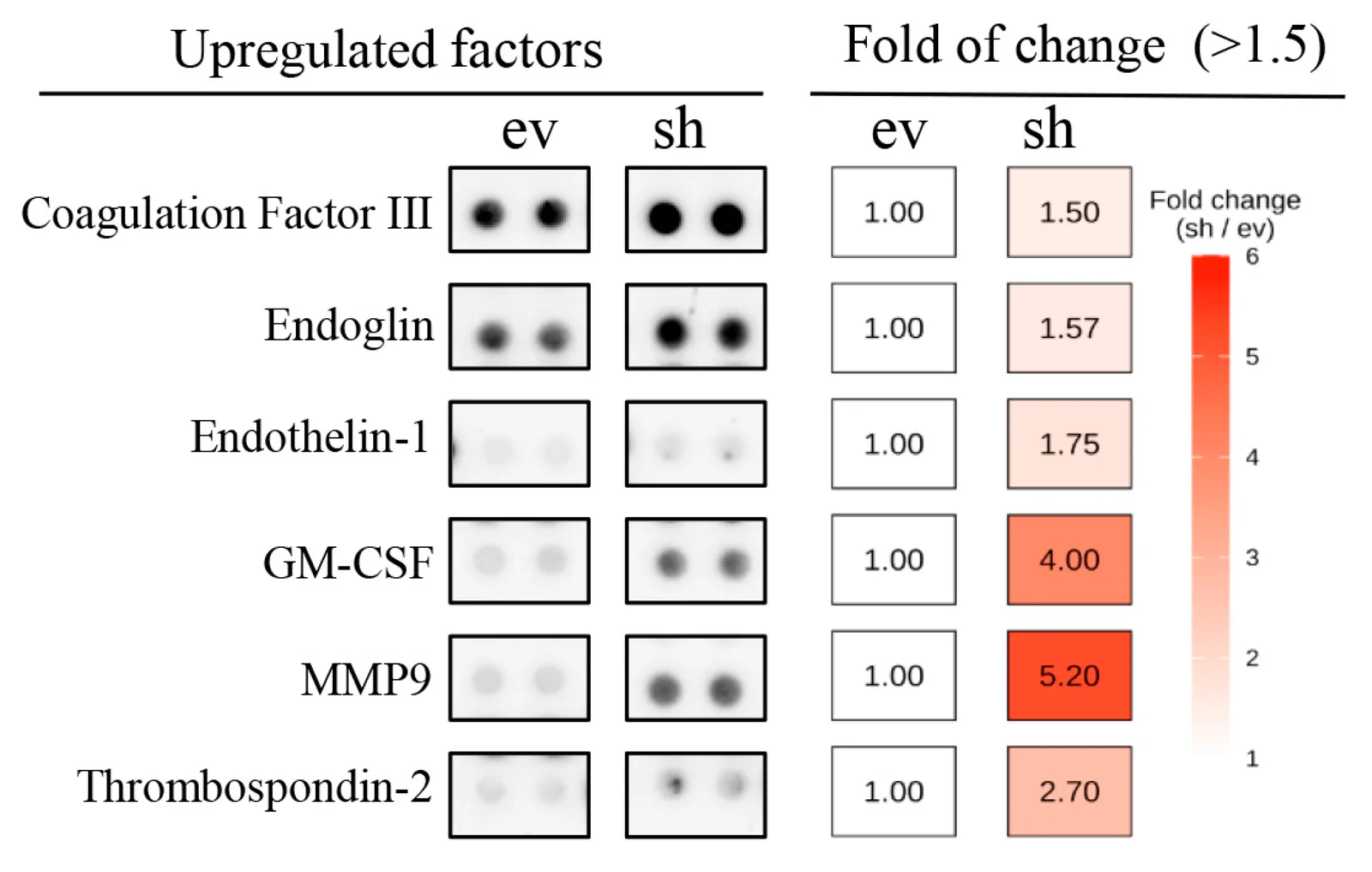
PDCD10 knockdown in GBM cells led to an increase in angiogenesis secretory profile. Culture media from empty vector-transduced T98G cells (evT98G) and PDCD10 knockdown T98G cells (shT98G) were analyzed using a human angiogenesis protein array with duplicate antibody spots. Duplicate immunoreactive spots are shown for the factors upregulated by more than 1.5-fold based on the quantitative analysis of the optical intensity (OI) of the immunoreactive spots by ImageJ software. Fold change was calculated as the OI of the sh group relative to the ev group, which was set to 1.00. The color scale indicates fold of change in angiogenic factors in the medium from shT98G versus evT98G culture medium.

Based on the pronounced pro-angiogenic effects of PDCD10 knockdown observed in multiple in vitro models and the CAM model, we investigated potential underlying mechanisms using an angiogenesis protein array in culture media from evT98G and shT98G cells. The array identified six proteins that were increased by ≥1.5-fold in shT98G culture medium compared with evT98G culture medium (Figure 4, original blots are shown in Supplementary Figure S2): coagulation factor III (1.50-fold), endoglin (1.57-fold), endothelin-1 (1.75-fold), GM-CSF (4.00-fold), MMP-9 (5.20-fold) and thrombospondin-2 (2.70-fold). Together with our previous findings in shU87 cells [31], these data indicate that PDCD10 knockdown in GBM cells increases the release of pro-angiogenic factors.

### 3.5. Hypervascularization and Acquisition of Endothelial-like Features in PDCD10-Knockdown Xenografts Derived from a Mouse Model of GBM

**Figure 5:**
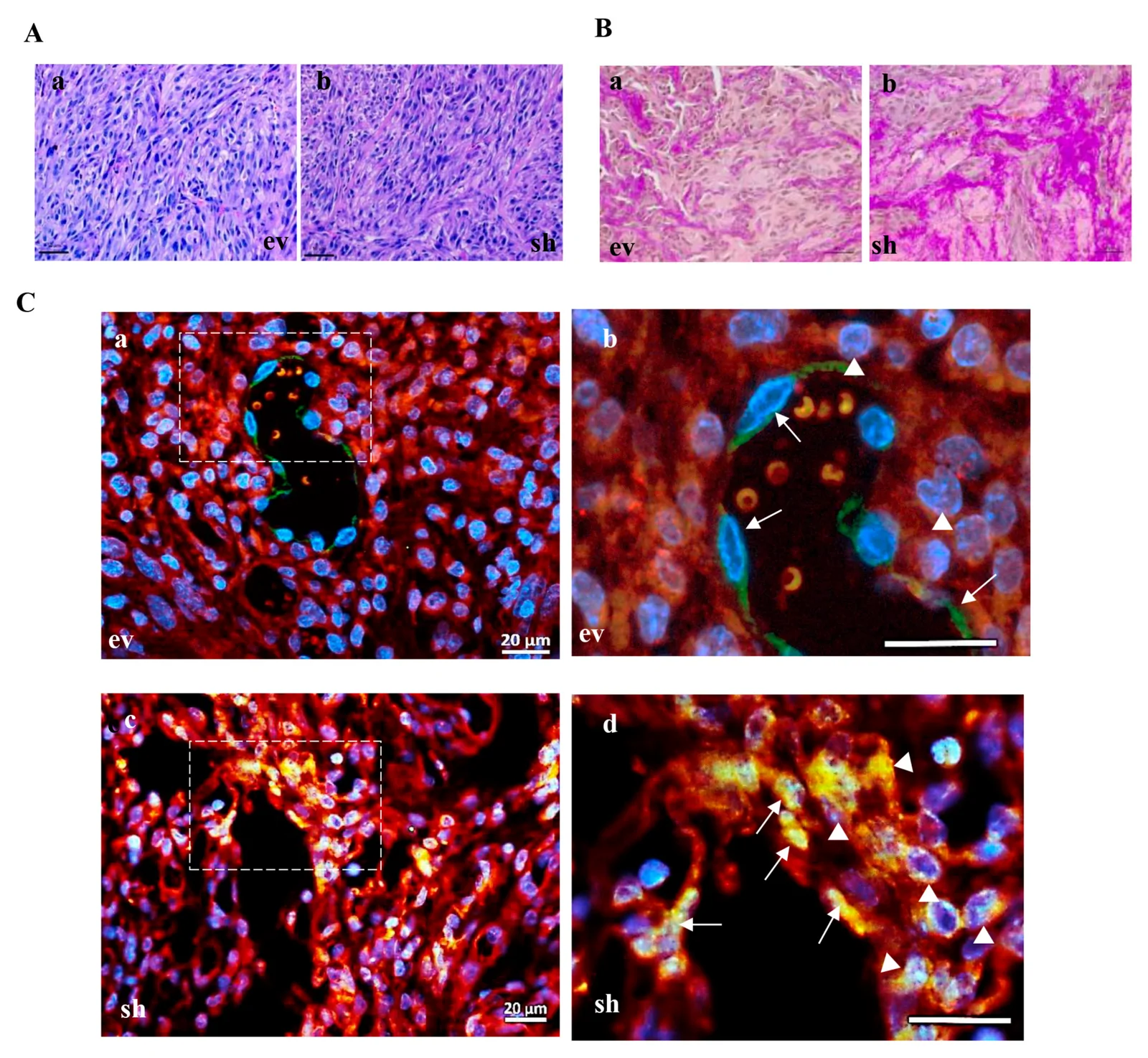
PDCD10 knockdown in GBM cells induces hypervascularization and endothelial-like features in a subcutaneous GBM xenograft model. Paraffin-embedded sections were obtained from a xenograft mouse model of GBM in which empty vector-transduced (ev) and PDCD10-knockdown (sh) U87 cells (ev/shU87) were subcutaneously implanted into the mouse flank. (**A**,**B**) Hypervascularization was visualized in shU87 xenograft tumor sections by H&E staining (**A**(**b**)) and Elastic van Gieson (EVG) staining (**B**(**b**)) compared to corresponding control (evU87) sections (**A**(**a**),**B**(**a**)). (**C**) Double immunofluorescence staining for the tumor cell reporter RFP (red) and CD31 (green) in xenograft sections. (**C**(**a**)) and (**C**(**c**)) show representative merged images of sections from evU87 and shU87 xenografts, respectively; (**C**(**b**)) and (**C**(**d**)) show enlarged views of the dashed box-marked fields in (**C**(**a**)) and (**C**(**c**)), respectively. In the control section (evU87), CD31 immunoreactivity was clearly detected in the endothelial cells without merging with GBM cell reporter RFP (arrows, (**C**(**b**))), suggesting that these CD31-positive endothelial cells represented host microvessels, whereas arrowheads indicate the positive RFP-stained GBM cells (**C**(**b**)), indicating no overlap between RFP and CD31. In contrast, overlapping signals of CD31 and tumor cell reporter RFP were observed in endothelial cells of some microvessels (arrows) and in a subset of GBM cells (arrowheads) in shU87 xenografts, indicating expression of endothelial marker in a subset of implanted shU87 cells (**C**(**d**)). Scale bar: 20 µm.

We previously reported that implantation of PDCD10-knockdown U87 cells promoted tumor growth in a mouse model of GBM [30,31]. The present study further examined archived xenograft sections derived from this model. H&E staining revealed more prominent microvessels and denser surrounding tumor cells in PDCD10-knockdown xenografts than in control tumors (Figure 5A). EVG staining showed enriched vascular and stromal features in shU87 sections (Figure 5B). These findings suggest that implanted PDCD10-knockdown GBM cells induced hypervascularity in the xenografts. To determine whether the abundant microvessels were host-derived or associated with implanted tumor cells, double immunofluorescence staining with red fluorescent protein (RFP) and CD31 was performed. Figure 5C(a) shows an overview of the control section (ev). In the enlarged view of the marked area of ev section (Figure 5C(b)), the immunoreactivity of CD31 was clearly detected in the endothelial cells (ECs) in a microvessel without merging with RFP (arrows). Since this CD31 antibody detects both human and mouse species, CD31 immunoreactivity detected here without overlapping with RFP suggests that these CD31-positive endothelial cells were most likely from mouse-derived host microvessels, whereas the positive RFP-stained cells representing implanted evU87 cells were negative for CD31 staining (arrowheads). Figure 5C(c) shows the overview image of the section in which multiple microvessels surrounded by dense implanted shU87 tumor cells are shown. Interestingly, a high-magnification view of the marked area (white box) in Figure 5C(c) clearly displays the overlapping expression of endothelial marker CD31 and GBM cell reporter RFP in the endothelial cells of some microvessels (arrows), as well as in a subset of shU87 GBM cells (arrowheads) (Figure 5C(d)), indicating that part of the implanted tumor cell population acquired CD31 expression. These findings are consistent with our previous reports [31] and suggest that PDCD10 knockdown is associated with endothelial marker acquisition in a subset of GBM cells within xenografts.

### 3.6. PDCD10-Knockdown GBM Cells Display Increased Plasticity

**Figure 6:**
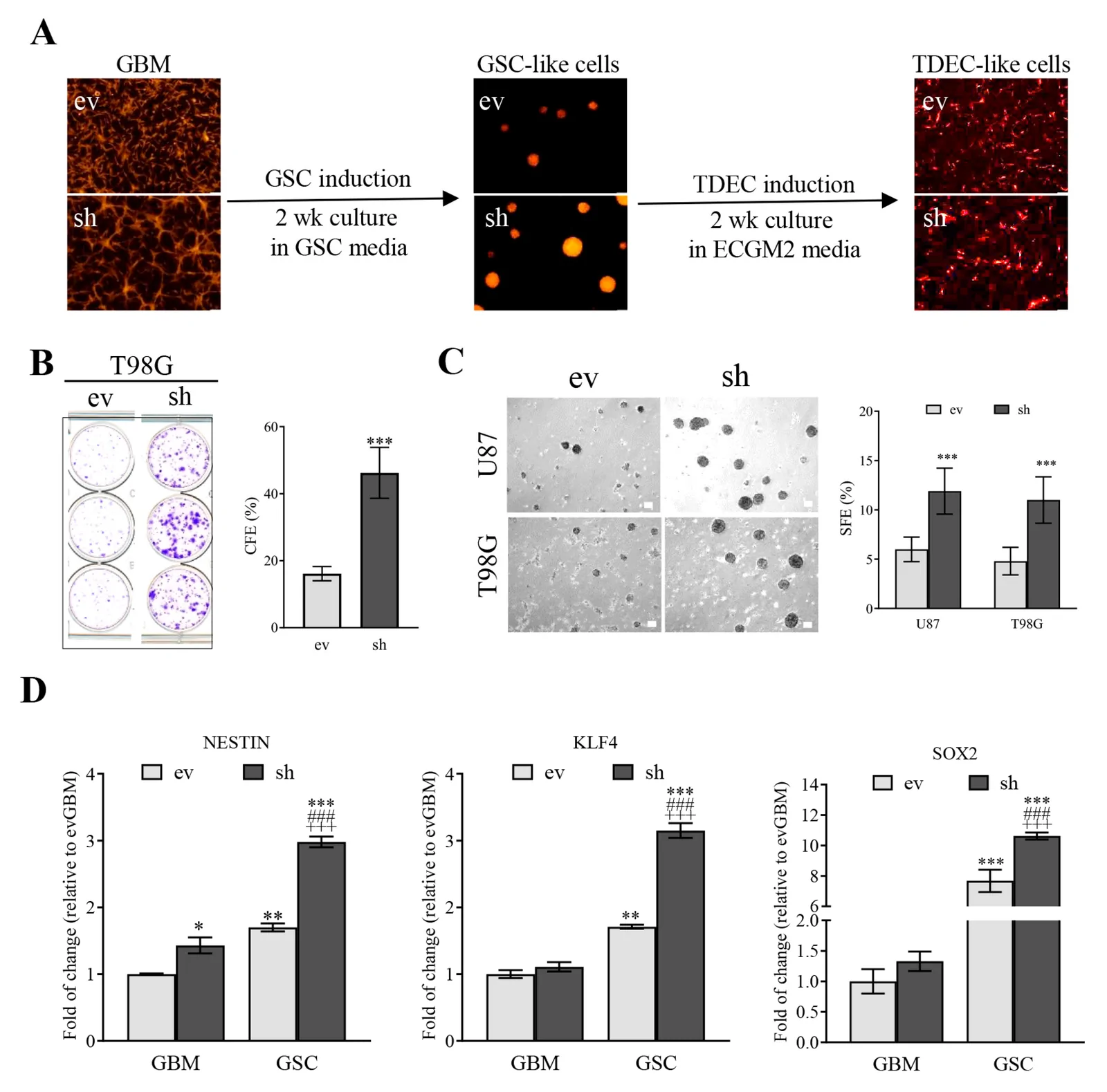
PDCD10 knockdown in GBM cells induced cell plasticity with glioma stem cell (GSC)-like characters. (**A**) Schematic illustration of the experimental workflow. Parental empty vector-transduced (ev) and PDCD10-knockdown (sh) U87 cells were cultured in stem cell culture medium for 2 wk to generate GSC-like cells. These GSC-like cells were then cultured in endothelial induction medium for 2 wk to generate TDEC-like cells. Characterization of GSC like cells is shown in panels (**B**–**D**), whereas characterization of TDEC-like cells is presented in Figure 7. (**B**) Colony formation assay. PDCD10 knockdown increased the clonogenic capacity of shT98G cells compared with the ev control. Representative crystal violet-stained colonies are shown on the left, and colony formation efficiency (CFE) is quantified on the right. ***, *p* < 0.001, compared with evT98G; (**C**) Neurosphere assay.PDCD10 knockdown in U87 and T98G cells increased sphere formation compared with the corresponding ev controls. Representative spheres are shown on the left, and sphere formation efficiency (SFE) is quantified on the right. (**D**) RT^2^-PCR analysis of the GSC-associated markers Nestin, KLF4 and SOX2 in parental ev/shU87 cells and their sphere-derived GSC-like cells. Expression of these markers in shGSC-like cells was significantly upregulated compared with parental cells (sh) and compared with the corresponding ev controls (evGSC). All experiments were reproduced in at least three independent experiments. *, *p* < 0.05, **, *p* < 0.01, ***, *p* < 0.001, compared with evU87; ###, *p* < 0.001, compared with shU87; +++, *p* < 0.001, compared with evGSC-like cells.

Based on the findings in the GBM xenograft sections, we examined whether PDCD10 knockdown enhanced stem-like plasticity in GBM cells. Empty vector-transduced (ev) and PDCD10-knockdown (sh) U87 parental cells were cultured in GSC medium to generate sphere-derived GSC-like cells (Figure 6A). The colony formation assay showed greater clonogenic capacity in shT98G cells than in evT98G controls. Colony formation efficiency increased by 46.2% in shT98G cells (*p* < 0.001; Figure 6B). Similar results were observed in ev and shPDCD10 LN229 cells (Supplementary Figure S3). In the neurosphere assay, shU87 and shT98G cells formed more and larger spheres than the corresponding ev controls. Sphere formation efficiency (SFE) increased approximately twofold in both PDCD10-knockdown cell lines (both *p* < 0.001; Figure 6C). We further examined the expression of GSC-associated markers. Nestin, KLF4 and SOX2 expression was significantly higher in shGSC-like cells than in shU87 parental cells and the corresponding evGSC-like controls (all *p* < 0.001; Figure 6D). These findings collectively support increased GSC-like plasticity in PDCD10-knockdown GBM cells under sphere-forming conditions.

### 3.7. Characterization of Tumor-Derived Endothelial-like Cells

**Figure 7:**
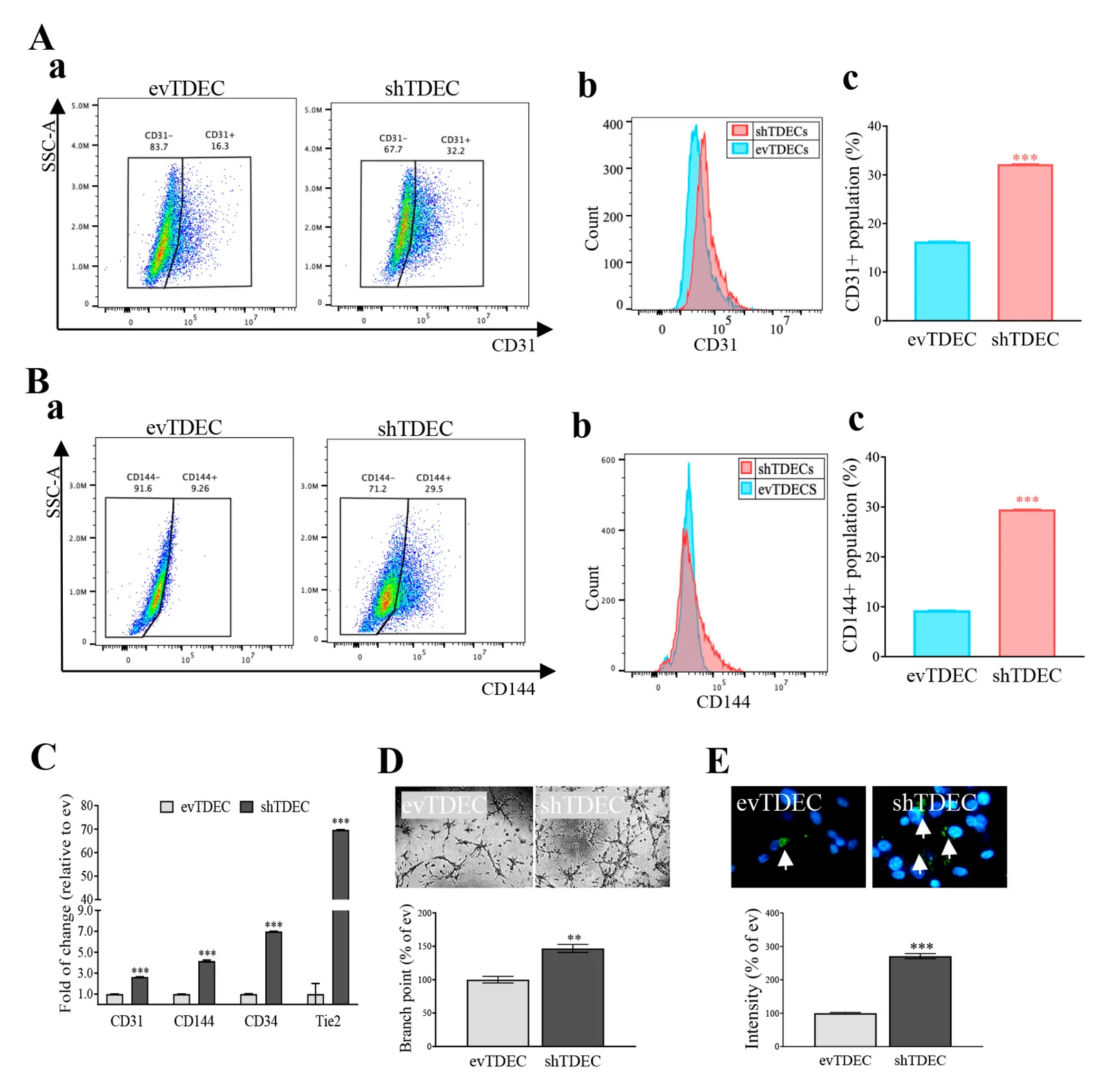
Characterization of TDEC-like cells. evGSC-like cells and shGSC-like cells were cultured in endothelial culture medium to generate evTDEC-like and shTDEC-like cells, respectively. These cells were harvested and subjected to analysis of the expression of multiple endothelial markers and to functional assays. (**A**,**B**) Flow cytometric analysis of CD31 (**A**) and CD144 (**B**) expression. (**A**(**a**)) and (**B**(**a**)) are representative dot plots showing the distribution of CD31- and CD144-positive cells, respectively, whereas (**A**(**b**)) and (**B**(**b**)) show the corresponding histograms presenting fluorescence-intensity distributions. (**A**(**c**)) and (**B**(**c**)) show quantitative analysis of CD31- and CD144-positive cells, respectively. (**C**) RT^2^-PCR analysis of CD31, CD144, CD34 and Tie2 expression in evTDEC-like cells and shTDEC-like cells. (**D**) Tube formation assay. Representative tube-like structures are shown in upper panel, and quantitative analysis of branching points from five random fields per well are shown in lower panel. (**E**) Acetylated low-density lipoprotein (Ac-LDL) uptake. Representative images show Ac-LDL-positive cells (arrows, upper panel). Fluorescence intensity was measured at excitation/emission wavelengths of 485/535 nm (lower panel). Experiments were repeated independently three times. **, *p* < 0.01, ***, *p* < 0.001 compared with evTDEC-like cells. For simplicity, TDEC in the figure denotes TDEC-like cells.

After generation and characterization of GSC-like cells from parental GBM cell spheres, next we continued to study TDEC-like cells derived from GSC-like cells as scheduled in the experimental working flow (Figure 6A). We checked the expression of CD31 and CD144 in TDEC-like cells by flow cytometry. Figure 7A shows a larger CD31-positive population in shTDEC-like cells than in evTDEC-like cells (Figure 7A(a)), accompanied by a rightward shift in fluorescence intensity (Figure 7A(b)). Similar results were obtained from CD144 assay (Figure 7B). Quantitative analysis indicated that the percentage of CD31-positive and CD144-positive cells increased approximately 2-fold and 3-fold, respectively, in shTDEC-like cells compared with the control evTDEC-like cells (both *p* < 0.001). We also checked the expression of multiple endothelial markers in TDEC-like cells by RT^2^-PCR analysis. The expression of CD31, CD144, CD34 and Tie2 was markedly upregulated by 2.6-, 4.2-, 7.0-, and 70.0-fold in shTDEC-like cells compared with evTDEC-like cells, respectively (all *p* < 0.001; Figure 7C). A significant upregulation of additional endothelial markers, including vWF and VEGFR2, was confirmed in shTDEC-like cells by RT^2^-PCR (in Supplementary Figure S4).

After assessing endothelial marker expression in TDEC-like cells, we further characterized their endothelial-like functions using tube-formation and Ac-LDL uptake assays. Compared with evTDEC-like cells, shTDEC-like cells formed more extensive tube-like networks and showed a significantly higher number of branching points (*p* < 0.001) (Figure 7D). Representative fluorescence images showed more intense Ac-LDL uptake in shTDEC-like cells than in evTDEC-like cells (Figure 7E, upper panel). Quantitative analysis demonstrated a 2.7-fold increase in Ac-LDL uptake in shTDEC-like cells compared with evTDEC-like cells (*p* < 0.001) (Figure 7E, lower panel).

## 4. Discussion

GBM is characterized by extensive and aberrant vascularization, a typical pathological feature closely associated with malignant progression [1,4]. Abnormal neo-angiogenesis supports rapid tumor cell growth and invasion and contributes to treatment resistance and tumor relapse [1,4,7]. Hypervascularization in GBM results from multiple processes, including vessel co-option, vasculogenic mimicry, tumor-secreted angiogenic mediators, host endothelial cells, and the perivascular stem cell niche [4,5,6,7,8]. In this context, tumor–endothelial interaction plays a fundamental role in neo-angiogenesis and malignant progression in GBM. The present study showed that PDCD10 deficiency in GBM cells was associated with an angiogenesis secretory profile, increased GSC-like properties and enhanced acquisition of endothelial-like molecular and functional signatures under endothelial induction conditions. These findings support complementary paracrine and cellular-plasticity processes through which PDCD10 loss may promote angiogenic activity in GBM.

PDCD10 is increasingly recognized as an important regulator of malignant tumors in a context-dependent manner [17]. This suggests that the functions of PDCD10 are determined by the molecular background and lineage-specific signaling networks of tumor cells. Our previous studies, ranging from analyses of patient samples to in vitro and in vivo models, support a tumor-suppressive role for PDCD10 in GBM [28,29,30,31,32]. In the present study, PDCD10 knockdown in GBM cells increased endothelial angiogenic activity in multiple in vitro assays and in the CAM model. Direct co-culture with shPDCD10 GBM cells increased endothelial proliferation, migration and invasion (Figure 2), whereas treatment of conditioned medium from shU87 and shT98G increased endothelial proliferation, adhesion, migration, invasion and tube formation (Figure 1). In the CAM model, shCM from T98G cells increased vascular branching and microvessel abundance (Figure 3). Angiogenesis array identified elevated levels of several angiogenesis factors in the medium derived from shT98G cells (Figure 4), consistent with our previous observations in shU87 cells [30]. The secretory profile of U87 and T98G cells were not identical, which may reflect genetic heterogeneity and lineage-specific signaling networks. GBM-derived soluble factors and tumor-endothelial crosstalk can promote endothelial activation, migration, tube-like organization and cell junction remodeling [7,37,38]. Thus, the altered secretory profile of shGBM cells may contribute to paracrine activation of endothelial cells through distinct signaling pathways driven by individual factors that collectively converge to exert synergistic pro-angiogenic effects. It has been shown that GSCs residing within specialized perivascular and hypoxic niches may actively reprogram the local microenvironment to drive pathological angiogenesis through multiple growth factors and angiogenic factors signaling networks. Therefore, we propose that the angiogenic factors upregulated in PDCD10 deficient condition may also act in an autocrine manner to reinforce GSC-like plasticity, thereby contributing to reprogramming angiogenesis. Further investigation of potential autocrine mechanisms and elucidation of the role of individual soluble factors in the pro-angiogenic effects of PDCD10 are warranted in the future.

We further examined archived sections from GBM xenografts generated by implantation of evU87 or shU87 cells [30,31]. shU87 xenografts showed increased vascular and stromal features (Figure 5A,B), consistent with the pro-angiogenic effects observed in the co-culture and CAM models. Interestingly, overlapping CD31 and GBM cell reporter RFP immunoreactivity was observed in a subset of RFP-positive PDCD10-knockdown cells (Figure 5C(d)). These double-positive cells displayed heterogeneous morphologies. Some retained an amoeboid tumor-cell morphology, whereas others showed an elongated endothelial-like morphology near microvessel walls. These observations suggest that PDCD10 deficiency potentially increases tumor cell plasticity and the acquisition of endothelial signatures. Given that these observations were derived from an archived subcutaneous xenograft GBM model, future studies using orthotopic models may provide a microenvironment for the interactions of implanted tumor cells with its native surrounding tissues, blood vessels, and organ-specific immune cells.

To further examine the plasticity of PDCD10-deficient GBM cells, we generated sphere-derived GSC-like cells from evU87 and shU87 cells (Figure 6A). PDCD10 knockdown increased colony formation (Figure 6B) and sphere formation efficiency (Figure 6C). These results indicate greater clonogenic and anchorage-independent growth under the tested conditions. shGSC-like cells also showed upregulation of the GSC-associated markers, including Nestin, KLF4 and SOX2, compared with shU87 parental cells and evGSC-like controls (Figure 6D). These findings support increased GSC-like plasticity after PDCD10 knockdown. Further study of PDCD10 deficiency-induced cell plasticity in patient-derived GBM cells may enhance the translational relevance.

In human GBM, GSC-like cells are closely integrated into the vascular microenvironment. They are enriched near tumor vessels and are maintained by endothelial niche-derived signals that support self-renewal and tumor propagation [8,38]. In turn, these cells can remodel the niche by producing angiogenic mediators and acquiring endothelial-like or pericyte-like properties [7,8,10,11,12,38]. It has been shown that GSCs act as the direct cellular parental cells to transdifferentiate into TDECs that contribute to neo-vascularization and eventually lead to therapy resistance in GBM [39,40]. We therefore examined whether PDCD10-knockdown sphere-derived GSC-like cells acquired endothelial-like characters under endothelial induction conditions. Notably, quantitative PCR revealed significant upregulation of multiple endothelial markers, including CD31, CD144, CD34, Tie2 (Figure 7C) and VEGFR2 and vWF (Figure S4) in shTDEC-like cells compared with evTDEC-like cells. Flow cytometry confirmed higher CD31-positive and CD144-positive populations in shTDEC-like cells (Figure 7A,B). Functional analyses provided evidence of more extensive formation of tube-like structures (Figure 7D) and enhanced Ac-LDL uptake (Figure 7E) in shTDEC-like cells. The coordinated acquisition of multiple endothelial markers, together with two characteristic endothelial functions, provides convergent evidence that shTDEC-like cells generated from PDCD10-knockdown GSC-like cells showed endothelial signatures under endothelial induction conditions (Figure 7). These results support an endothelial-like signature, consistent with previous reports that GBM stem-like cells can acquire endothelial-like features and contribute to tumor vascularization [10,11]. Further validation of these findings using patient-derived GSCs, applying lineage-tracing approaches, and performing functional vessel incorporation studies in vivo would substantially strengthen the findings obtained from the present study. In addition, although the use of two independent shRNA systems for knockdown of PDCD10 supports reproducibility in the present study, the potential shRNA-mediated off-target effects cannot be completely excluded. A rescue strategy applying gene technique to restore PDCD10 expression in shPDCD10 cells may be helpful to address this issue in the future.

## 5. Conclusions

**Figure 8:**
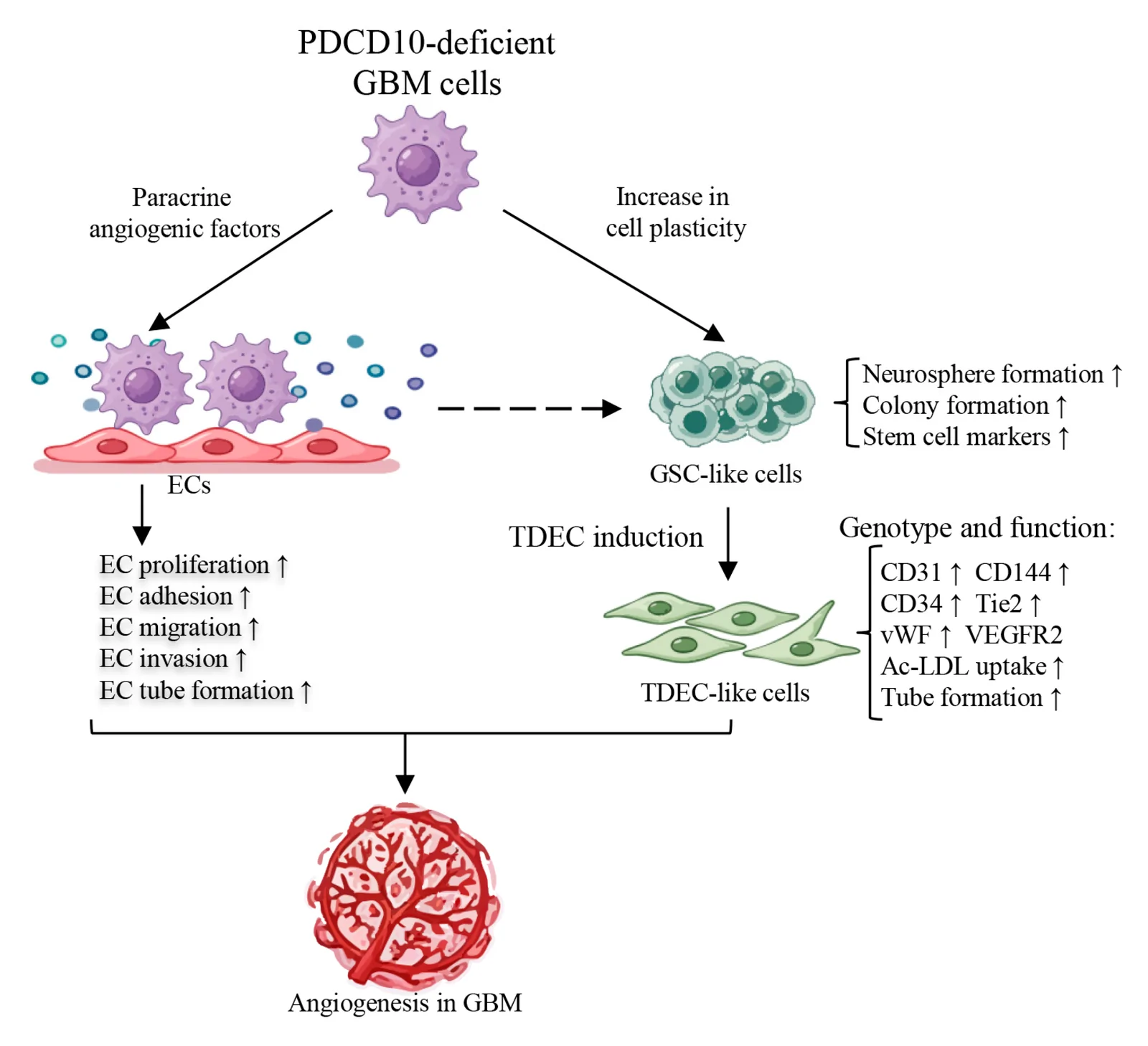
Proposed model of angiogenic reprogramming induced by PDCD10 deficiency in GBM cells. PDCD10 deficiency promotes neo-angiogenesis in GBM through two complementary mechanisms. First, PDCD10-deficient GBM cells secrete pro-angiogenic factors that facilitate paracrine crosstalk with endothelial cells (ECs), resulting in endothelial activation. Second, PDCD10 loss enhances GBM cell plasticity, as demonstrated by increased clonogenicity, sphere-forming capacity, and expression of GSC-associated markers. The altered secretory profile may also potentially contribute to the reprogramming of GBM cells toward GSC-like and TDEC-like states (dashed arrow). These TDEC-like cells acquire molecular signatures and functional characters of endothelial cells. The paracrine angiogenic factors and increased cellular plasticity resulting from PDCD10 deficiency cooperate to drive tumor angiogenesis in GBM.

Taken together, the major findings are schematically illustrated in Figure 8. This study identified for the first time, to our knowledge, dual paracrine and cell plasticity mechanisms underlying PDCD10 deficiency-mediated angiogenic activity, highlighting PDCD10 as a potential therapeutic target against neo-angiogenesis in GBM.

## Figures and Tables

**Table 1:** List of primers and corresponding annealing temperatures for RT^2^-PCR in the present study. (for: forward sequence; rev: reverse sequence)

Primer Name	Sequence	Annealing Temperature (°C)
PDCD10		60
for	TGGCAGCTGATGATGTAGAAG	
rev	TCGTGCCTTTTCGTTTAGGT	
NESTIN		60
for	CTCCAAGAATGGAGGCTGTAGGAA	
rev	CCTATGAGATGGAGCAGGCAAGA	
KLF4		60
for	GGCTGCGGCAAAACCTACAC	
rev	CGGGCGAATTTCCATCCAC	
SOX2		60
for	ACCGGCGGCAACCAGAAGAACAG	
rev	GCGCCGCGGCCGGTATTTAT	
CD31		60
for	TTCCTGACAGTGTCTTGAGTGG	
rev	GCTAGGCGTGGTTCTCATCT	
CD144		60
for	CACAGGCACGTCACACTGA	
rev	GCTGATGCAAACAGAGCACC	
CD34		60
for	GACCCTGATTGCACTGGTCA	
rev	AATAAGGGTCTTCGCCCAGC	
Tie2		60
for	ATGGCAGGGGCTCCAGTGTGA	
rev	TGGAGCACTGTCCCATCCGGC	
VEGFR2		60
for	CTCTTGGCCGTGGTGCCTTTG	
rev	GTGTGTTGCTCCTTCTTTCAAC	
vWF		60
for	CCGATGCAGCCTTTTCGGA	
rev	TCTGGAAGTCCCCAATAATCGAG	
GAPDH		60
for	TCACCACCATGGAGAAGGC	
rev	GCTAAGCAGTTGGTGGTGCA	
RPS13		60
for	CGAAAGCATCTTGAGAGGAACA	
rev	TCGAGCCAAACGGTGAATC	

## Data Availability

The original contributions presented in this study are included in the article/Supplementary Materials. Further inquiries can be directed to the corresponding author.

## Supplementary Materials

The following supporting information can be downloaded at https://www.mdpi.com/article/10.3390/cells15171532/s1, Figure S1: Original immunoblots for Figure 1B. Original immunoblots showing PDCD10 expression in empty vector-transduced (ev) and PDCD10-knockdown (sh) U87 (A) and T98g (B) cells; Figure S2: Original immunoblots of angiogenesis array. Human angiogenesis antibody array was performed by incubation of the membrane pre-coated with 55 specific antibodies with conditioned medium CM) derived from empty vector-transduced (ev) and PDCD10-knockdown (shPDCD10) T98g cells. Blue rectangles indicate the upregulated angiogenesis factors presented in Figure 4. Number 1 to 6 respectively refer to Coagulation Factor III, Endoglin, Endothelin-1, GM-CSF, MMP-9 and Thrombospondin-2; Figure S3: PDCD10 knockdown enhanced colony formation in LN229 cells. Colony formation assay was performed in LN229 cells that received lentiviral-mediated transduction of either empty vector (ev) or PDCD10 plasmid (sh). Representative images (left) and quantitative analysis (right) showed increased colony formation in shLN229 cells compared with that from evLN229. Data are presented as the mean ± SD from at least three independent experiments. CFE: colony formation efficiency. ***, *p* < 0.001, compared with ev; Figure S4: The expression of vWF and VEGFR2 in TDEC-like cells. Expression of vWF and VEGFR2 in evTDEC-like (ev) and shTDEC-like (sh) cells was analyzed by RT^2^-PCR. Data are presented as mean ± SD from three independent experiments. ***, *p* < 0.001, compared with evTDEC-like cells.
